# Twist1 Transcriptional Targets in the Developing Atrio-Ventricular Canal of the Mouse

**DOI:** 10.1371/journal.pone.0040815

**Published:** 2012-07-16

**Authors:** Pavle Vrljicak, Rebecca Cullum, Eric Xu, Alex C. Y. Chang, Elizabeth D. Wederell, Mikhail Bilenky, Steven J. M. Jones, Marco A. Marra, Aly Karsan, Pamela A. Hoodless

**Affiliations:** 1 Terry Fox Laboratory, British Columbia Cancer Agency, Vancouver, Canada; 2 Department of Medical Genetics, University of British Columbia, Vancouver, Canada; 3 Michael Smith Genome Sciences Centre, British Columbia Cancer Agency, Vancouver, Canada; 4 Department of Pathology and Laboratory Medicine, University of British Columbia, Vancouver, Canada; IRCCS-Policlinico San Donato, Italy

## Abstract

Malformations of the cardiovascular system are the most common type of birth defect in humans, frequently affecting the formation of valves and septa. During heart valve and septa formation, cells from the atrio-ventricular canal (AVC) and outflow tract (OFT) regions of the heart undergo an epithelial-to-mesenchymal transformation (EMT) and invade the underlying extracellular matrix to give rise to endocardial cushions. Subsequent maturation of newly formed mesenchyme cells leads to thin stress-resistant leaflets. TWIST1 is a basic helix-loop-helix transcription factor expressed in newly formed mesenchyme cells of the AVC and OFT that has been shown to play roles in cell survival, cell proliferation and differentiation. However, the downstream targets of TWIST1 during heart valve formation remain unclear. To identify genes important for heart valve development downstream of TWIST1, we performed global gene expression profiling of AVC, OFT, atria and ventricles of the embryonic day 10.5 mouse heart by tag-sequencing (Tag-seq). Using this resource we identified a novel set of 939 genes, including 123 regulators of transcription, enriched in the valve forming regions of the heart. We compared these genes to a Tag-seq library from the *Twist1* null developing valves revealing significant gene expression changes. These changes were consistent with a role of TWIST1 in controlling differentiation of mesenchymal cells following their transformation from endothelium in the mouse. To study the role of TWIST1 at the DNA level we performed chromatin immunoprecipitation and identified novel direct targets of TWIST1 in the developing heart valves. Our findings support a role for TWIST1 in the differentiation of AVC mesenchyme post-EMT in the mouse, and suggest that TWIST1 can exert its function by direct DNA binding to activate valve specific gene expression.

## Introduction

The transformation of the heart tube into a four-chambered organ divided by valves and septa is a critical event during mammalian heart development and is required for proper function. Initially, the embryonic heart is a linear tube composed of endocardial and myocardial cell layers separated by an acellular extracellular matrix (ECM) termed the cardiac jelly. During formation of the heart valves and septa, the cardiac jelly, which gives rise to the endocardial cushions [1], begins to accumulate in the region between the atria and ventricles, known as the atrio-ventricular canal (AVC), and the junction between the ventricles and the major arteries, known as the outflow tract (OFT). At around embryonic day (E) 9 in the mouse and day 26 in humans, inductive signals from the myocardium mediated by members of the Notch, Wnt and TGFβ pathways activate endocardial cells in these regions to undergo an epithelial-to-mesenchymal transformation (EMT) [2]–[6]. EMT is a multi-step process in which polarized and adhesive endocardial cells transform into non-polarized and highly motile mesenchyme cells [7]. During EMT, groups of endocardial cells destined to undergo transformation are first specified and become hypertrophic. These cells then lose polarity markers and intercellular cadherins and adherens junctions. Finally degradation of the basement membrane by matrix metalloproteinases (such as MMP2) and cell delamination driven by reorganization of the cytoskeleton leads to the migration and invasion of EMT-generated cells into the endocardial cushion. After invasion of the ECM, the newly transformed mesenchyme cells proliferate to expand the forming cushions into the lumen [8] and undergo differentiation into fibroblastic valve interstitial cells [9]. Removal of excess mesenchyme cells by apoptosis starts at E12.5 in mice, and patterning of ECM molecules relative to direction of blood flow then turns the endocardial cushions into thin stress-resistant AV valve leaflets and semilunar valve cusps by the first week after birth [10]–[12].

The process of endocardial cushion transformation demonstrates tight temporal and spatial gene expression specificity controlled by a network of transcription factors. Single-gene studies have helped to determine the important role of a number of transcription factors during this process, including SOX9 [13], SNAI2 [14] and β-CATENIN [3]. In addition to their role in heart valve development, these factors are also critical in many embryonic tissues that undergo an EMT (e.g. [15]), and have been implicated in cancer metastatic progression, which involves a type of EMT (e.g. [16]–[19]).

*Twist1*, a member of the basic helix-loop-helix (bHLH) family of transcription factors, is highly expressed in the mesenchymal cells of the endocardial cushions of the AVC and OFT, with expression peaking at E10.5 before decreasing at E12.5 [20], [21]. Similar to the above factors, *Twist1* over-expression is associated with enhanced metastasis as it promotes cell survival and cell invasion in transformed cells (e.g. [22]). Research in chick AVC development has suggested a role for TWIST1 in the promotion of proliferation and migration of endocardial cushion cells, coupled with inhibition of their differentiation [23]. Subsequent over-expression studies in mouse confirmed a role for TWIST1 in proliferation and regulation of ECM protein expression [24], indicating that TWIST1 might have a role in endocardial cushion cell maturation post-EMT. Interestingly, research in *Twist1* null mice has not revealed an obvious AVC endocardial cushion defect prior to death at about E11 [25] although defects in neural crest cell contribution to the OFT have been reported [26].

Since there may be underlying molecular changes in the *Twist1* null AVC that may indicate defects not manifested in the embryo prior to death, in this study, we used tag sequencing (Tag-seq) to identify gene expression changes associated with loss of *Twist1* in the AVC. Tag-seq, a type of Digital Gene Expression (DGE) analysis termed elsewhere as SAGE-Seq, is a technique that combines Serial Analysis of Gene Expression (SAGE) with massively parallel sequencing [27] to produce short 21-base sequence tags representing the expressed RNA transcripts. By mapping the sequence tags to transcriptome and genome databases, the originating transcript can be identified. Moreover, since the data is digital, the frequency of a tag can be quantified and compared between genes and tissues. Tag-seq is independent of previous transcript knowledge and can be used for novel gene and transcript variant discovery. We first generated Tag-seq libraries from atria, ventricles, AVC and OFT of the mouse heart at E10.5 and used these libraries to identify a high-confidence AVC- and OFT-enriched gene set containing novel endocardial cushion genes and transcription factors. We then used this set to evaluate changes observed in the AVC of *Twist1* mutant embryos by Tag-seq. Our data on the gene expression differences between *Twist1* mutant and wild-type AVC at E10.5, coupled with chromatin immunoprecipitation, suggests that TWIST1 plays a critical role in mesenchymal differentiation post-EMT in the mouse, by directly regulating expression of AVC-enriched genes.

## Materials and Methods

### Ethics Statement

All mice procedures were performed at the animal facility of the British Columbia Cancer Agency according to protocols approved by the University of British Columbia Animal Care Committee and following Canadian Council on Animal Care guidelines.

### Tissue Collection

E10.5 embryos (Theiler stage 17) were collected from timed-matings of C57BL/6J females and the hearts were manually dissected using 30½G needles to separate the various regions of the heart. Blood was removed by puncturing the heart chambers and washing the tissue with PBS. All tissue was collected in TRIzol reagent (Invitrogen). Images of dissected heart regions can be found in Figure S1. Tissue from 20 embryos was collected for the atria and ventricle libraries while tissue from 25 embryos was collected for the AVC and OFT. 8 AVCs were collected from *Twist1* null AVC. Tissue for each region was pooled to avoid possible bias due to individual variation and to obtain at least 400 ng of total RNA for Tag-seq library construction. After isolation, RNA quality was assessed using an Agilent Bioanalyzer. *Twist1* null mice have been described previously [25] and were maintained on an ICR (Taconic Farms) background.

### Gene Expression Analysis

Tag-seq libraries were constructed as described [27], using at least 400 ng of DNase I-treated total RNA. Briefly, after double-stranded cDNA synthesis with oligo(dT) beads (Invitrogen), the cDNA was digested with an anchoring restriction enzyme (NlaIII) and ligated to an Illumina specific adapter (Adapter A) containing a recognition site for the Type IIS tagging enzyme MmeI. Following MmeI (New England Biolabs) digestion, which cuts 20-bp from the recognition site, a second Illumina adapter (Adapter B) that contains a 2-bp degenerate 3′ overhang was ligated. Tags flanked by both adapters were enriched by PCR, and the PCR products were run on a 12% PAGE gel, excised and purified. Cluster generation and sequencing was performed on the Illumina cluster station and analyzer (Illumina) following the manufacturer's instructions. Raw sequences were extracted from the resulting image files using Bustard 1.8.28 and processed with ELAND (Illumina). Reads were assessed for quality using the Chastity filter (Illumina) with a threshold of 0.6. The tags were generated based on the first 21 bases of each read, which corresponds to the transcript-derived tag sequence. The first 4 bases are always the NlaIII site. The Tag-seq data is available at Gene Expression Omnibus [28] through the accession number GSE37746.

Tag-seq data was analyzed and mapped to genes using DiscoverySpace v4.0 [29]. Background tags, tags that could not be mapped to the genome, and those that contained 1-bp mismatch from tags generated from highly expressed genes were excluded. For all analysis, we selected tag-types with greater than 5 tags per library (tags with 5 or fewer counts in a library were considered not expressed) and used only unambiguous sense mappings based on the RefSeq database (http://www.ncbi.nlm.nih.gov/RefSeq/). All tags representing a single gene were summed into an overall expression count to account for transcript variants. All tag counts were normalized to library size and values represented as tags per million. Differential expression analysis was done using the Bioconductor package edgeR [30]–[32] using the exact test in the package to calculate the fold changes and p-values based on a dispersion value of 0.12. Gene Ontology analysis was performed with DAVID [33], [34]. Enrichment was calculated against the whole RefSeq database as a background group and p-values were generated to represent one-tail Fisher Exact Test statistics.

Reverse transcription followed by quantitative PCR (RT-qPCR) and *in situ* hybridization was described previously [35]. Primers for qPCR and generation of *in situ* hybridization probes are found in Table S1. *Periostin* and *Tbx20* probes were described previously [36], [37].

### Chromatin Immunoprecipitation

Chromatin immunoprecipitation was carried out as described [38] using either an anti-TWIST1 antibody (Abcam, ab50887, lot#772574) or a mouse non-specific IgG (Sigma, F2883) followed by sequencing (ChIP-seq) or qPCR (ChIP-qPCR). Briefly, whole heart or limb samples were homogenized and fixed in 1% formaldehyde for 10 minutes prior to the addition of glycine to 0.125 M. Fixed cells were washed with PBS and lysed for 15 minutes in ChIP cell lysis buffer (10 mM Tris–HCl, pH 8.0, 10 mM NaCl, 3 mM MgCl_2_, 0.5% NP-40) followed by resuspension in ChIP nuclear lysis buffer (1% SDS, 5 mM EDTA, 50 mM Tris–HCl, pH 8.1). After 30 minutes on ice, cells were sonicated on ice-water (S3000 Ultrasonic Cell Disruptor Processor, Fisher) for 20 cycles of 30 seconds on, 30 seconds off. After removal of cellular debris by centrifugation, chromatin was precleared with Protein A/G UltraLink Resin (20 µl, ThermoFisher 53135) in fresh ChIP dilution buffer (0.01% SDS, 1.1% Triton X-100, 167 mM NaCl, 16.7 mM Tris–HCl, pH 8.1). Beads were spun down and removed and 3 µg of antibody was added to supernatants containing the sonicated DNA. All buffers contained protease inhibitor cocktail tablets (Roche). Following overnight incubation at 4°C, samples were incubated with fresh Protein A/G beads for 4 h rotating at 4°C. The chromatin-bound beads were precipitated and washed in low salt buffer (0.1% SDS, 1% Triton X-100, 2 mM EDTA, 20 mM Tris–HCl, pH 8.1, 150 mM NaCl), high salt buffer (low salt buffer with 500 mM NaCl), lithium chloride buffer (0.25 M LiCl, 1% NP-40, 1% deoxycholate, 1 mM EDTA, 10 mM Tris–HCl, pH 8.1) and twice with TE buffer. Antibody-chromatin complexes were eluted twice from the beads with 125 µl elution buffer (1% SDS, 0.1 M NaHCO_3_) each time. The collected samples were incubated with 1.0 µl Proteinase K (20 mg/ml) (Invitrogen) and 2.5 µl RNaseA (10 mg/ml Invitrogen) overnight at 65°C to reverse cross-link. Chromatin was purified by phenol–chloroform extraction and ethanol precipitation and resuspended in 50 µl dH_2_O.

For ChIP-seq, DNA from three pooled ChIP samples (from 49, 107 and 85 whole hearts corresponding to 16.2 µg, 54.5 µg and 33.5 µg of chromatin respectively) was purified by SDS–PAGE to obtain 100–300 bp fragments for sequencing on Illumina 1G. Input DNA from the same E10.5 heart tissue was purified and sequenced for a control library. Sequences were mapped to NCBI Build 36 (mm9) reference mouse genome using Burrows-Wheeler-Aligner (BWA) v0.5.x [39]. Peaks were identified using FindPeaks 3.1 [40]. To identify TWIST1 binding sites, peaks were thresholded at a minimum peak height of 10 based on a false discovery rate of 0.01. To further limit false positives, for each peak that passed the FDR threshold, the coverage of the peak was compared to that of the control sample in the region +/−400 bp. Two criteria were set: the local z-score (calculated for each peak based on the peak height and the control coverage) had to be greater than 1.8, and the fold change between the TWIST1 peak and the control peak had to be greater than 1.75.

For ChIP-qPCR, the fold enrichment of each target site was calculated as 2 to the power of the cycle threshold (cT) difference between the IgG immunoprecipitated sample and the TWIST1 immunoprecipitated sample. Primers used for ChIP-qPCR are listed in Table S1.

## Results

### AVC and OFT Share Significant Gene Expression

Since expression of *Twist1* is spatially restricted to the endocardial cushions in both the AVC and OFT of the developing heart, we first assessed the diversity of genes that are normally enriched in the AVC and OFT. We constructed Tag-seq gene expression libraries from the atria, ventricles, AVC and OFT of E10.5 mouse hearts (Table 1). Each library was sequenced to a minimum depth of 7 million tags for a total of over 34 million tags. To exclude very low abundance transcripts and tags that might have been generated by library construction or sequencing errors, we only included tags that were represented more than 5 times in a library. The high quality tags corresponded to 10,670 RefSeq genes expressed (Table S2). We examined 16 genes previously characterized as enriched in the AVC and involved during its development to establish whether our Tag-seq libraries captured known patterns of gene expression (Table S3). Expression of these genes in the AVC Tag-seq library spanned two orders of magnitude ranging from 13 tags per million for the transcription factor *Snai2* to 1,473 tags per million for the structural molecule *Vimentin*. Most of the genes we examined were significantly differentially expressed (p-value<0.05) in the AVC when compared to the atria and ventricles. Furthermore, AVC enrichment (calculated by edgeR as the normalized fold-change of AVC expression over the atria and ventricle libraries) ranged from just under two-fold for *Postn* to 303-fold for the transcription factor *Sox4*, and indicated that the Tag-seq libraries represent a reliable source of gene expression information over a wide dynamic range.

**Table 1:** Overview of Tag-seq libraries.

Library ID	Description*	All Tags	HQ Tags**	HQ Tag-types
MM0265	E10.5 Atria	8,303,915	4,666,514	35,110
MM0263	E10.5 Atrio-ventricular canal	10,445,112	6,075,421	58,407
MM0266	E10.5 Ventricles	8,284,532	4,223,810	32,822
MM0264	E10.5 Outflow tract	7,072,418	5,267,618	56,744
	**Total**	**34,105,977**	**20,233,363**	**101,847**
MM0513	E10.5 *Twist1* ^−/−^ Atrio-ventricular canal	17,262,266	12,977,237	79,271

* Atria, atrio-ventricular canal, ventricles and outflow tract were isolated from E10.5 (Theiler stage 17) mouse hearts for Tag-seq library construction. **High quality (HQ) tag-types were present at greater than 5 tags per library.

**Figure 1:**
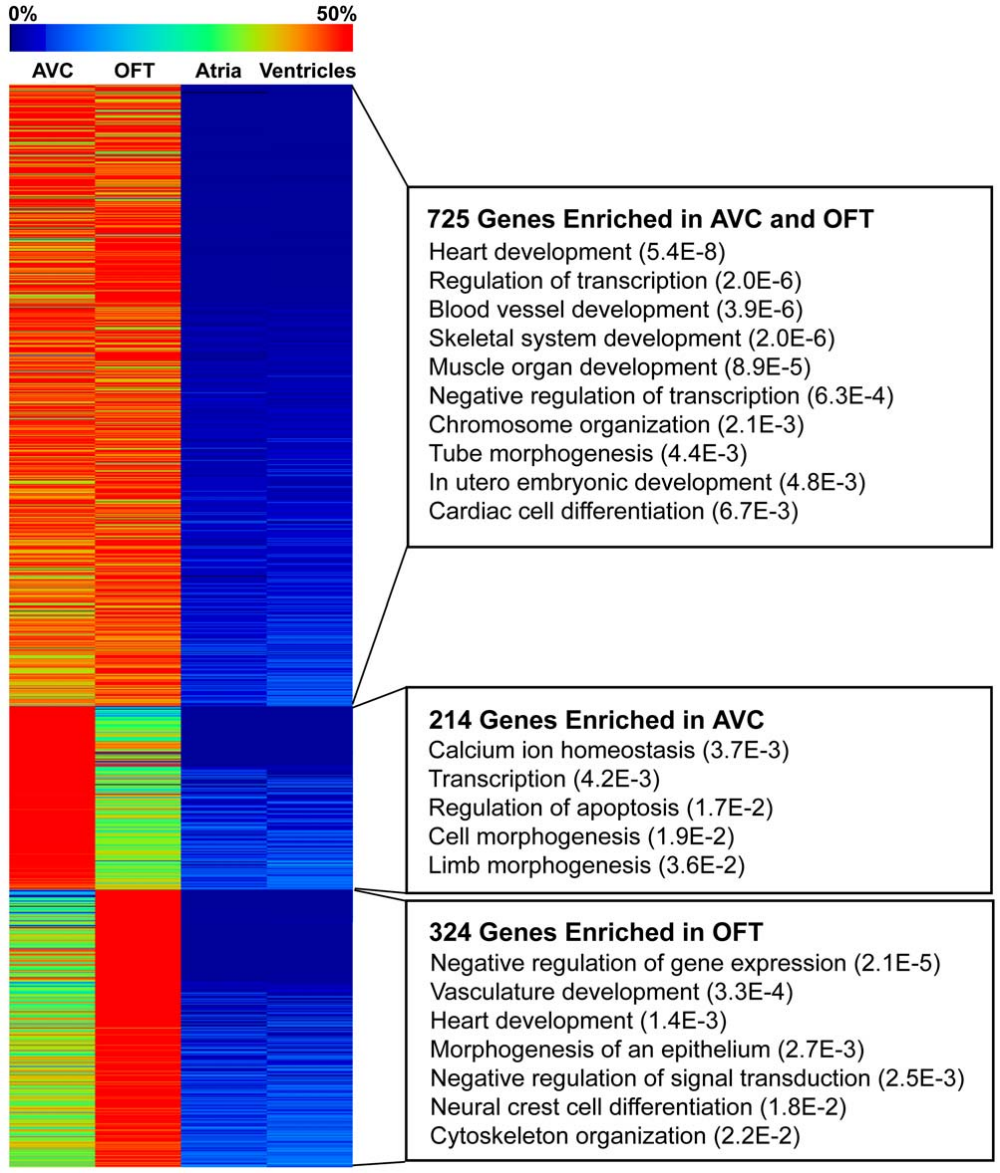
AVC and OFT shared gene expression. Most genes enriched in the AVC were also enriched in the OFT. The heatmap shows expression levels across E10.5 heart Tag-seq libraries normalized per gene for the overlapping genes with significant enrichment (p-value<0.05) in the AVC and OFT or genes enriched in only AVC or only OFT. Expression of all tag-types mapping in the sense direction to the same RefSeq gene were pooled and all tag counts were normalized as tags per million reads sequenced. Expression fold changes (enrichment) and associated p-values were calculated by doing an exact test using edgeR. Select enriched Gene Ontology (GO) categories are shown with Fisher Exact Test p-values in parentheses. See Table S5 for complete GO analysis results.

Since the AVC and OFT both undergo a similar process of endocardial cushion formation and EMT, we speculated that genes critical for early valve formation would be more highly expressed in these regions as compared to the atria and ventricles. Comparison of the 939 genes enriched in the AVC (p-value<0.05) against the 1049 genes enriched in the OFT (p-value<0.05) revealed that 725 of the genes are common between the two (Figure 1 and Table S4A). Genes with shared AVC and OFT enrichment included known endocardial cushion development genes such as *Bmp2*, *Jag1*, *Gata4*, and *Sox9*. The majority of the genes in our AVC- and OFT-enriched list have not been described in the context of endocardial cushion development. Furthermore, a number of genes specific to the OFT or AVC were also identified, such as *Galanin* and *Adamts19* in the AVC and *Rgs5* and *Gabra4* in the OFT (Table 2, Figure S2, and Tables S4B and S4C).

**Table 2:** Selected Genes Enriched in the AVC and/or OFT.

						AVC over A&V		OFT over A&V	
Gene symbol	RefSeq accession	AVC	OFT	Atria	Ventricles	Fold Change	P-Value	Fold Change	P-Value
**A. Genes enriched in both AVC and OFT**									
**Transcriptional regulators**									
*Twist1*	NM_011658	918.0	299.1	63.4	55.9	8.16	1.36E-04	2.85	4.90E-02
*Sox9*	NM_011448	514.4	376.7	4.1	4.3	63.08	2.66E-09	49.33	1.09E-08
*Aes*	NM_010347	232.4	304.8	0.0	3.3	67.67	3.31E-08	94.49	3.31E-09
*Gata4*	NM_008092	227.4	122.8	9.4	5.5	15.90	1.13E-05	9.19	5.16E-04
*Zeb1*	NM_011546	185.4	196.1	6.9	7.8	13.16	4.56E-05	14.87	1.53E-05
*Tead2*	NM_011565	120.0	96.9	7.3	10.9	6.91	1.53E-03	5.96	4.00E-03
*Gata6*	NM_010258	102.2	101.6	6.6	20.6	3.95	1.82E-02	4.20	1.61E-02
*Sox4*	NM_009238	98.8	86.5	0.0	0.0	303.66	1.08E-06	272.38	2.18E-06
*Gata5*	NM_008093	78.2	66.5	5.4	3.8	8.79	1.25E-03	7.99	2.55E-03
*Tbx20*	NM_194263	51.7	40.1	1.5	0.0	29.95	3.00E-04	24.68	9.46E-04
*Gata3*	NM_008091	40.1	99.1	0.0	1.7	21.42	1.13E-03	55.91	3.54E-06
*Klf4*	NM_010637	32.1	47.8	0.0	0.0	99.34	1.81E-04	150.97	1.38E-05
*Tbl1x*	NM_020601	24.2	19.9	0.0	0.0	75.13	8.36E-04	63.43	2.02E-03
*Tle2*	NM_019725	23.7	8.9	0.0	0.0	73.60	8.36E-04	28.92	3.91E-02
*Hes6*	NM_019479	18.6	41.2	0.0	0.0	57.98	2.78E-03	130.26	3.39E-05
*Lef1*	NM_010703	18.1	11.0	0.0	0.0	56.45	2.78E-03	35.51	1.68E-02
*Gata2*	NM_008090	14.2	39.7	0.0	0.0	44.50	7.80E-03	125.55	4.10E-05
**ECM structural molecules and modifiers**									
*Col3a1*	NM_009930	1442.0	2334.3	94.9	108.7	7.52	2.01E-04	13.01	4.50E-06
*Hspg2*	NM_008305	123.0	134.9	9.6	5.7	8.37	6.29E-04	9.81	2.93E-04
*Col9a3*	NM_009936	120.5	38.2	3.0	0.0	38.39	3.83E-06	13.03	3.53E-03
*Mmp14*	NM_008608	89.3	127.2	0.0	0.0	274.56	2.18E-06	400.07	1.42E-07
*Col5a1*	NM_015734	65.1	92.2	5.4	8.1	5.06	1.29E-02	7.65	1.80E-03
*Tnc*	NM_011607	47.7	523.1	3.6	0.0	12.81	1.32E-03	148.73	4.76E-11
**Signalling molecules**									
*Igfbp5*	NM_010518	1563.9	1149.7	49.7	17.5	24.62	6.32E-08	19.35	3.73E-07
*Bmp2*	NM_007553	219.6	27.1	4.5	0.0	48.25	1.48E-07	6.41	1.60E-02
*Bmper*	NM_028472	173.5	303.9	10.9	1.7	14.32	2.73E-05	26.78	4.03E-07
*Htra1*	NM_019564	70.3	31.9	0.0	0.0	216.36	1.08E-05	101.08	1.44E-04
*Csnk1e*	NM_013767	43.6	36.3	0.0	0.0	134.56	3.39E-05	114.89	7.51E-05
*Rspo3*	NM_028351	39.9	43.1	0.0	0.0	123.23	5.00E-05	136.22	2.81E-05
*Jag1*	NM_013822	12.0	27.9	0.0	0.0	37.76	1.68E-02	88.53	2.93E-04
**Proliferation**									
*Ccnd2*	NM_009829	594.3	1117.7	66.9	77.7	4.36	5.63E-03	8.77	7.88E-05
*Cdca7*	NM_025866	114.1	157.7	3.0	6.6	12.20	1.34E-04	17.99	1.26E-05
**B. AVC-specific genes**									
*Gal*	NM_010253	38.2	0.0	0.0	0.0	118.02	6.11E-05	1.00	1.00E+00
*Adamts19*	NM_175506	31.2	5.5	0.0	0.0	96.58	2.30E-04	18.26	1.69E-01
*Ryr3*	NM_177652	11.4	2.7	0.0	0.0	35.92	1.68E-02	9.47	5.28E-01
**C. OFT-specific genes**									
*Isl1*	NM_021459	2.3	72.9	1.5	0.0	1.51	1.00E+00	44.71	2.74E-05
*Rgs5*	NM_009063	1.5	41.0	0.0	0.0	5.60	1.00E+00	129.63	3.39E-05
*Gabra4*	NM_010251	1.0	24.3	0.0	0.0	4.06	1.00E+00	77.24	6.35E-04
*Lamc2*	NM_008485	0.0	15.6	0.0	0.0	1.00	1.00E+00	49.94	5.45E-03
*Frzb*	NM_011356	2.6	13.3	0.0	0.0	8.96	5.28E-01	42.73	1.13E-02

Expression levels represent all sense tags for a gene and are shown as tags per million.

AVC- or OFT- fold change as calculated by Bioconductor package edgeR. P-values<0.05 are considered enriched.

Gene Ontology analysis of the genes enriched in both the AVC and OFT revealed significant enrichment for transcriptional regulation and embryonic developmental processes (Figure 1 and Table S5), which supported our previous finding that at E10.5 there is increased signaling and transcription factor activity [20]. Among the 123 transcriptional regulatory genes enriched in both the AVC and OFT, members of the GATA family (*Gata2*, *3*, *4*, *5* and *6*) were particularly well represented. GATA3, GATA4 and GATA6 have each been shown to play important roles during endocardial cushion development leading to AVC and OFT defects when mutated in mice [41]–[43]. In contrast, GATA2 and GATA5 have not previously been described in the context of endocardial cushion development. Other transcriptional regulators not previously identified in the context of valve and septa formation included *Klf4*, *Zeb1* (an inhibitor and an inducer of EMT, respectively), and *Tbl1x* (a mediator of Wnt signaling). Importantly, the bHLH transcription factor *Twist1* was the most highly-expressed, DNA-binding, transcription factor in the AVC at E10.5. As noted above, no AVC phenotype has been reported in the *Twist1* null mouse.

### *Twist1* Null AVC Shows Dramatic Gene Expression Changes

**Figure 2:**
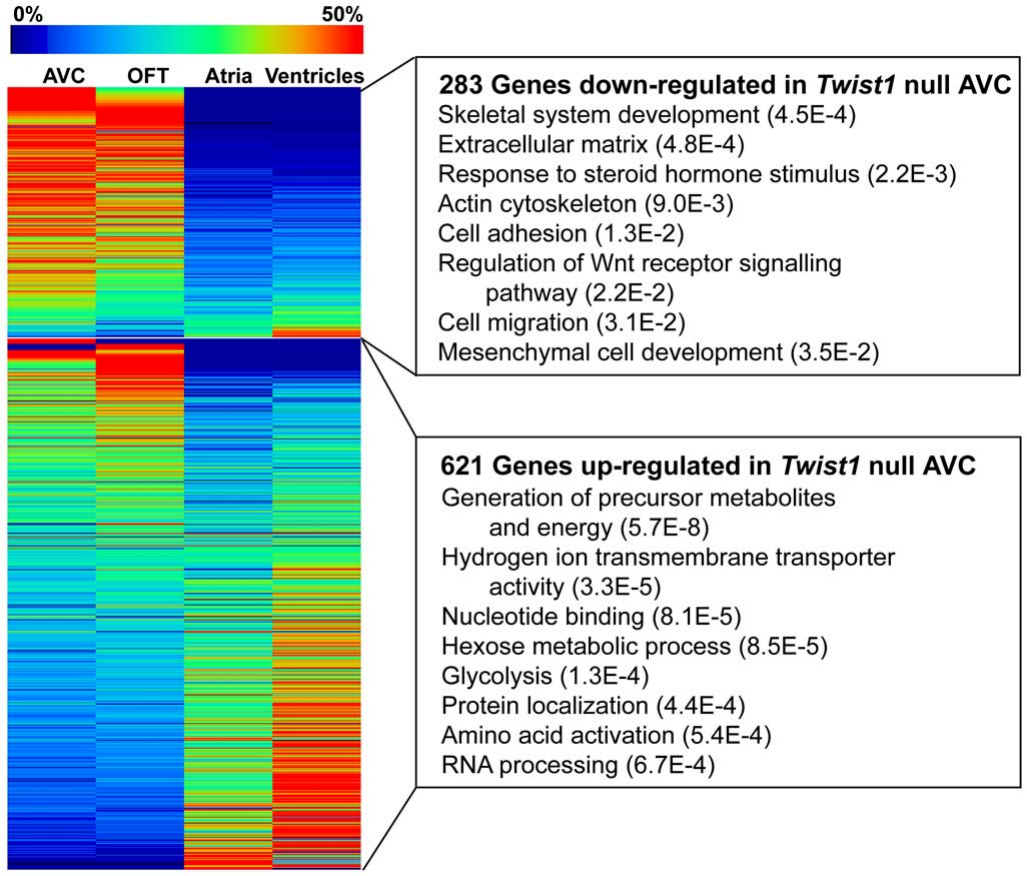
Altered gene expression in *Twist1* null AVC. Genes significantly down-regulated (p-value<0.05) in the *Twist1* null AVC are enriched in the wild-type AVC and OFT, while genes up-regulated in *Twist1* null AVC tend to be enriched in the atria and ventricles. Expression of all tag-types mapping in the sense direction to the same RefSeq gene were pooled and all tag counts were normalized as tags per million reads sequenced. Expression fold changes (enrichment) and associated p-values were calculated by doing an exact test using edgeR. Select enriched Gene Ontology (GO) categories are shown with Fisher Exact Test p-values in parentheses. See Table S7 for complete GO results.

To study the role of TWIST1 in the context of AVC development, we analyzed the genetic changes in mice lacking a functional copy of the *Twist1* gene [25] by creating a Tag-seq library from E10.5 AVC. Comparison of the *Twist1* null and wild-type AVC libraries identified 283 genes significantly down-regulated (p-value<0.05) in the *Twist1* null AVC and 621 genes significantly up-regulated (Figure 2 and Table S6). Of note, 119 (42.0%) of the genes down-regulated in the *Twist1* null AVC were also enriched in the wild-type AVC library. In contrast, only 7 genes (1.1%) up-regulated in the *Twist1* null AVC were enriched in the wild-type AVC library while 254 (40.9%) up-regulated genes were enriched in the atria and ventricles libraries (depleted in the wild-type AVC).

We used Gene Ontology (GO) to further analyze the genes down-regulated in the *Twist1* null heart (Table 3, Figure 2 and Table S7). Consistent with a proposed role of TWIST1 in ECM remodeling [24], genes down-regulated in the *Twist1* null AVC were significantly enriched for ECM proteins (p-value = 4.8E-4), such as *Col3a1*, *Col6a2* and *Decorin*. Some of these genes, such as *Col1a1*, *Mgp, Papss2* and *Biglycan*, are also involved in skeletal system development (also an enriched GO category, p-value = 4.5E−4), which is consistent with the known role of TWIST1 in controlling differentiation of bone and cartilage [44], [45].Most of these genes were also enriched in the wild-type AVC libraries. *Periostin*, another ECM protein and a proposed direct target of TWIST1 in osteoblasts [46], was also reduced significantly in the *Twist1* null AVC (4.2 fold-change, p-value = 1.1E−2). Other targets of TWIST1 suggested in chick endocardial cushion cells [23], *Cdh11, Mmp2* and *Tbx20,* showed decreased expression levels in the *Twist1* null library but the reductions were not significant (Table 3). This suggests that TWIST1 may not be a major regulator of these genes in the mouse AVC and more likely acts as a modulator in combination with other AVC-specific transcription factors.

**Table 3:** Selected gene expression changes in *Twist1* null AVC.

							*Twist1* over WT AVC	
Gene symbol	RefSeq accession	AVC	OFT	Atria	Ventricles	*Twist1* ^−/−^	Fold Change	P-Value
**A. Genes down-regulated in ** ***Twist1*** ** null AVC**								
**Transcriptional regulators**								
*Twist1*	NM_011658	918.0	299.1	63.4	55.9	4.6	0.006	8.49E-13
*Snai1*	NM_011427	43.5	18.4	2.1	1.4	4.8	0.137	2.92E-03
*Sox9*	NM_011448	514.4	376.7	4.1	4.3	113.5	0.263	1.09E-02
*Klf4*	NM_010637	32.1	47.8	0.0	0.0	4.5	0.174	1.22E-02
*Sox4*	NM_009238	98.8	86.5	0.0	0.0	19.6	0.238	1.32E-02
*Sox13*	NM_011439	27.0	29.6	0.0	1.4	3.9	0.180	1.50E-02
*Klf7*	NM_033563	13.7	17.3	0.0	0.0	1.5	0.147	4.80E-02
**ECM components**								
*Dcn*	NM_007833	60.4	41.8	33.6	33.9	4.7	0.097	4.42E-04
*Col3a1*	NM_009930	1442.0	2334.3	94.9	108.7	206.9	0.171	8.77E-04
*Bgn*	NM_007542	49.4	36.9	0.0	0.0	4.6	0.116	1.37E-03
*Tnc*	NM_011607	47.7	523.1	3.6	0.0	4.9	0.127	3.25E-03
*Col1a1*	NM_007742	160.2	204.2	24.9	19.7	26.2	0.196	3.79E-03
*Col1a2*	NM_007743	234.7	239.1	126.4	111.1	48.0	0.244	9.51E-03
*Col6a2*	NM_146007	18.4	8.4	4.9	1.7	2.9	0.199	3.79E-02
**Bone, cartilage and tendon development**								
*Papss2*	NM_011864	218.9	115.5	0.0	0.0	12.0	0.066	7.42E-06
*Mgp*	NM_008597	12.3	1.9	2.6	1.7	0.0	0.022	7.80E-03
*Bmp2*	NM_007553	219.6	27.1	4.5	0.0	51.2	0.278	1.70E-02
*Bmp1*	NM_009755	57.8	63.2	5.1	2.1	14.7	0.306	4.52E-02
**Cell migration**								
*Tns1*	NM_027884	56.1	30.1	0.0	0.0	9.5	0.205	1.25E-02
*Kitl*	NM_013598	121.3	160.0	1.5	1.7	27.4	0.270	1.90E-02
*Efnb1*	NM_010110	74.1	73.1	9.4	3.3	15.9	0.258	2.33E-02
*Nrg1*	NM_178591	7.9	16.3	0.0	0.0	0.0	0.033	3.91E-02
**Apoptosis**								
*Daxx*	NM_007829	63.1	126.9	11.6	4.5	9.8	0.188	7.25E-03
*Tnfrsf12a*	NM_013749	12.5	17.3	0.0	0.0	1.2	0.133	2.45E-02
*Ccar1*	NM_009810	195.4	171.4	7.9	7.3	57.4	0.350	5.00E-02
**Other: Validated by ChIP**								
*Tpm4*	NM_001001491	45.5	57.9	0.0	0.0	5.1	0.138	4.14E-03
*Dagla*	NM_198114	13.2	4.8	1.3	0.0	1.2	0.126	2.45E-02
*9030425E11Rik*	NM_133733	16.3	9.1	0.0	0.0	1.9	0.153	2.83E-02
*Tdrd7*	NM_146142	53.8	37.2	20.6	31.0	6.0	0.137	2.83E-03
*Ror2*	NM_013846	46.2	17.1	13.3	20.6	7.3	0.193	9.90E-03
*4930402H24Rik*	NM_029432	19.1	18.4	1.7	3.6	2.5	0.168	3.03E-02
*Chd9*	NM_177224	29.8	14.4	6.2	3.6	5.9	0.242	5.04E-02
*Iffo2*	NM_183148	11.5	12.5	2.4	2.4	2.2	0.245	8.41E-02
*Nin*	NM_008697	9.4	4.6	1.9	6.6	1.6	0.225	1.52E-01
*Tgfbi*	NM_009369	3.5	9.7	0.0	0.0	0.0	0.072	2.92E-01
*Glrb*	NM_010298	4.4	2.3	3.9	5.9	0.6	0.211	4.40E-01
*Eif3h*	NM_080635	4.1	1.7	0.0	0.0	0.6	0.225	4.40E-01
**B. Genes up-regulated in ** ***Twist1*** ** null AVC**								
*Gatsl2*	NM_030719	0.0	2.3	0.0	0.0	6.7	30.338	3.91E-02
*Wdr75*	NM_028599	5.8	20.3	12.0	15.4	74.1	14.561	4.39E-05
*Sik1*	NM_010831	6.6	16.1	10.7	17.5	61.6	10.702	2.94E-04
*Abra*	NM_175456	1.0	11.4	0.0	2.4	8.5	8.164	6.59E-02
*Tpcn1*	NM_145853	15.6	19.1	1.9	2.1	79.1	5.945	2.42E-03
**C. Putative TWIST1 targets from literature (as identified in chick endocardial cushions** [23]								
*Postn*	NM_015784	144.1	138.6	52.5	32.9	29.0	0.241	1.07E-02
*Mmp2*	NM_008610	194.2	237.2	26.8	29.6	105.8	0.648	4.03E-01
*Cdh11*	NM_009866	55.1	33.4	31.1	35.7	33.7	0.729	5.84E-01
*Tbx20 variant 1*	NM_194263	51.7	40.1	1.5	0.0	43.3	0.996	1.00E+00
*Tbx20 variant 2*	NM_020496	14.5	14.8	1.3	2.1	9.0	0.743061	7.90E-01

A critical step in EMT is the acquisition of an invasive phenotype by the newly formed mesenchyme cells. Consistent with a role of TWIST1 in controlling the invasion and cell migration phenotype following initial transformation, genes down-regulated in the *Twist1* null AVC included kit ligand *(Kitl)*, the focal adhesion protein *Tns1,* and the ErbB2/3 ligand *Nrg1*. During metastatic progression, TWIST1 is thought to prevent the death of newly transformed cells by inhibiting apoptosis [47], [48]. In addition to their role in cell migration, *Kitl* and *Nrg1* are negative regulators of apoptosis. Other regulators of apoptosis down-regulated in the *Twist1* null AVC library include *Daxx*, *Ccar1*, and the tumor necrosis factor receptor superfamily, member 12A (*Tnfrsf12a*).

Interestingly, several noteworthy transcription factors are down-regulated in the *Twist1* null AVC, suggesting that TWIST1 is acting as part of a transcriptional network. Three members of the SRY family of transcription factors, *Sox9, Sox4* and *Sox13,* are both down-regulated in the *Twist1* null library and enriched in the AVC. Zinc finger transcription factors, including *Snai1* and two members of Kruppel-like factor family (*Klf4* and *Klf7*), were also down-regulated. *Twist1* and *Snai1* are commonly over-expressed in tissues undergoing EMT, have been shown to have genetic interactions in regulating gene expression of ECM proteins (e.g. [49], [50]), and their combined mutation enhances abnormal cranial suture fusion [51].

Of the 621 genes that were up-regulated in the *Twist1* null AVC, GO analysis indicated that many are involved in metabolism and energy production. These included genes critical in the electron transport chain and many of the essential enzymes in glycolysis (Table S7B).

**Figure 3:**
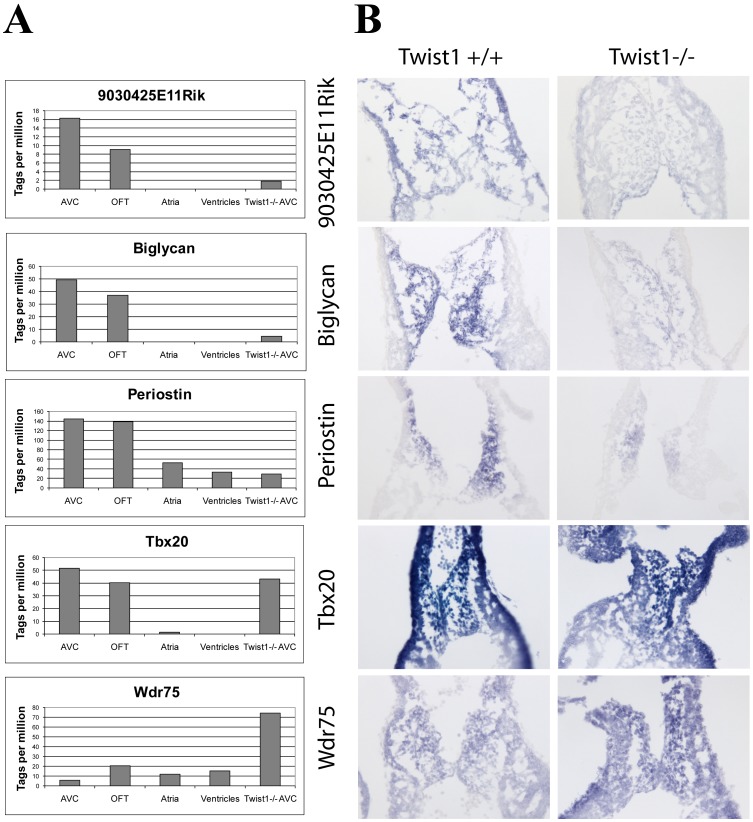
Regulation of AVC gene expression by TWIST1. A. Gene expression changes in *Twist1* null AVC by Tag-seq. Expression of all tag-types mapping in the sense direction to the same RefSeq gene were pooled and all tag counts were normalized as tags per million reads sequenced. Expression of *9030425E11Rik*, *Biglycan*, and *Periostin* are down-regulated in *Twist1* null AVC. *Wdr75* expression is up-regulated in *Twist1* null AVC, while *Tbx20* expression is not significantly changed. B. Gene expression changes in *Twist1* null AVC are confirmed by *in situ* hybridization.

To verify the quality of our expression results, and to study the regional distribution of genes up- and down-regulated in the *Twist1* null AVC, we examined the expression of selected genes by *in situ* hybridization analysis (Figure 3). Although the expression domains of the genes were preserved, we found that the ECM molecules *Periostin* and *Biglycan* showed a marked drop in expression in the AVC mesenchymal compartment in the *Twist1* null embryos. By Tag-seq analysis the normalized fold-changes were established as 4.2 and 8.6-fold lower in the null AVC respectively. Similarly, the RIKEN gene 9030425E11 (a putative orthologue of the tight junction protein *CLMP*) was 6.5-fold down-regulated in the *Twist1* null AVC. The WD repeat containing gene *Wdr75* was up-regulated in the null AVC as predicted by our Tag-seq data (fold-change of 14.6). In contrast, *Tbx20,* which showed very little expression change in the *Twist1* null AVC Tag-seq library (no fold change in transcript variant 1 and 1.3 down-regulation in transcript variant 2), had no detectable change in gene expression by *in situ* hybridization.

Overall, our gene expression analysis indicates that TWIST1 activity is necessary to establish proper AVC gene expression following EMT.

### TWIST1 Directly Regulates AVC Gene Expression

**Figure 4:**
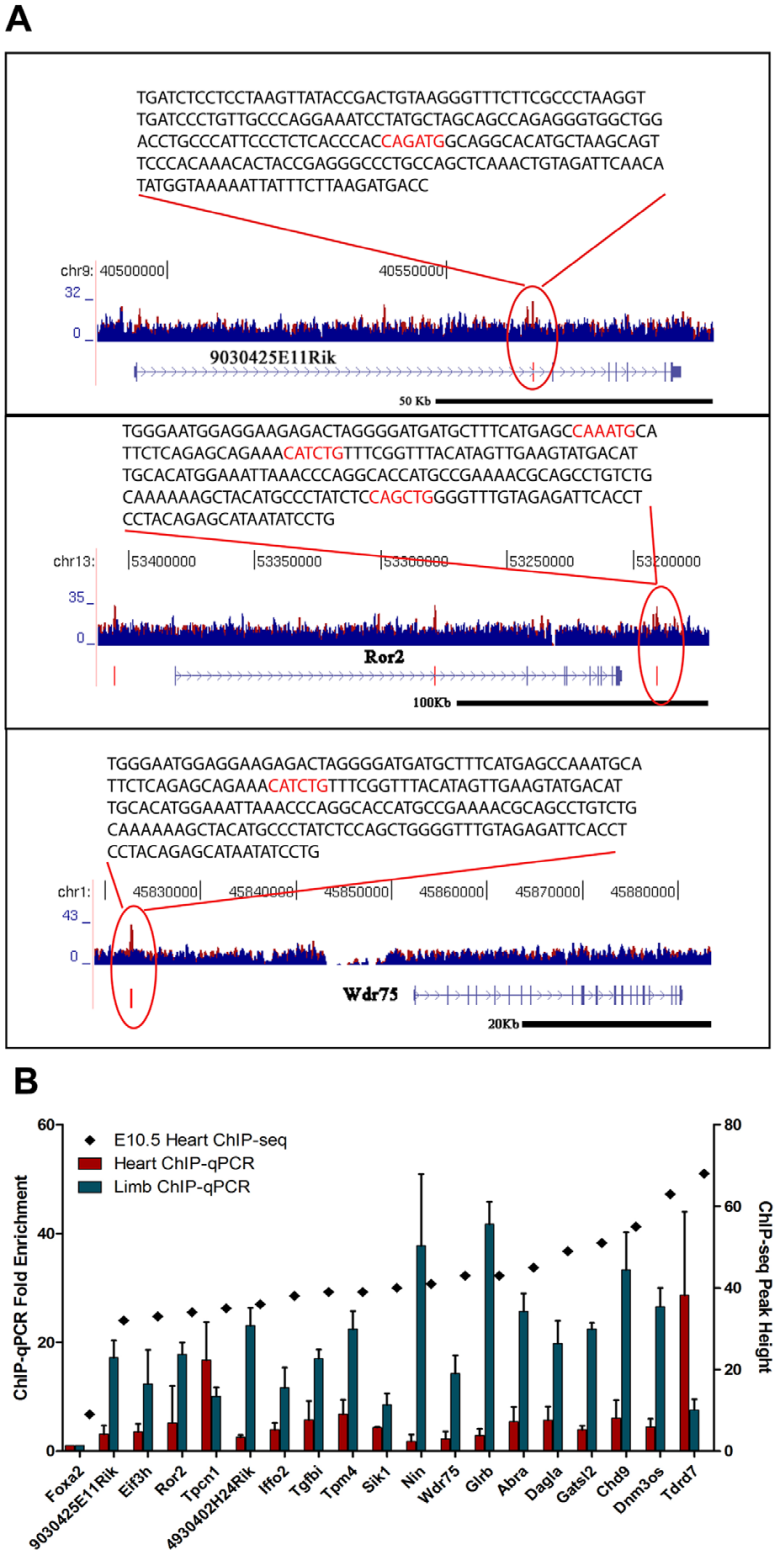
TWIST1 regulates AVC expression by directly binding to DNA. A. Genomic location of ChIP-seq peaks showing TWIST1 binding sites. ChIP-seq results for TWIST1 (red) and input DNA control (blue) are shown over a selected genomic window. The location of the E-box sequence within the ChIP-seq peak is highlighted. B. TWIST1 binding was confirmed by ChIP-qPCR using primers in Table S1. Enrichment was calculated as 2 to the power of the cycle threshold (cT) difference between the IgG immunoprecipitated sample and the TWIST1 immunoprecipitated sample. *DNM3os* was used as a positive control and a region of the *Foxa2* promoter sequence as a negative control for TWIST1 binding. All enrichment values were normalized to the negative control to account for variability between ChIPs. The mean and standard deviation of the enrichment from three ChIP replicates are shown with the height of the peak established by ChIP-seq.

Typically, dimerization of TWIST1 with a ubiquitously expressed bHLH factor (E-protein, such as E12) ensures the DNA-binding domain binds an E-box sequence (3′CANNTG5’) [52]. DNA binding usually leads to the activation of gene expression; however, TWIST1 can also act as a negative regulator of gene expression by direct interaction with the basic domain of other bHLHs or by sequestering E-proteins [53], [54]. To test whether the gene expression changes observed in the *Twist1* null AVC were a result of direct binding of TWIST1 to DNA, we performed chromatin immunoprecipitation followed by massively parallel sequencing (ChIP-seq) using anti-TWIST1 antibodies on E10.5 heart tissue. The resulting sequence reads were aligned to the mouse genome to create peaks that identify regions of TWIST1 binding activity, which were compared with peaks generated from an input DNA control from the same tissue (Figure 4). The 9038 (height 10 or greater) TWIST1 ChIP-seq peaks that were absent from the control library were assigned to 9745 different genes using GREAT (great.stanford.edu/). Due to the complexity of the library, in this study, we limited our analysis to peaks with a height of 30 or greater that were associated with genes expressed in the AVC. This focused our analysis on 520 peaks associated with 647 genes. 57 of these genes with high confidence TWIST1 binding sites were differentially expressed in the *Twist1* null AVC by Tag-seq (26 were down-regulated, 31 were up-regulated; p-value<0.05; Table S8).

These novel putative targets of TWIST1 represented a variety of functions critical for cell differentiation. For example, the TWIST1 target *Chd9* is a chromatin modifier and a regulator of transcription, while *Eif3h* is an elongation factor involved in gene translation. *Dagla* is a diacylglycerol lipase involved in neuronal cell proliferation and function. Finally, *Iffo2* is an intermediate filament and *Tgfbi* is an ECM component and modifier that causes a postnatal growth defect and increased frequency of tumors when mutated in mice. Up-regulated genes bound by TWIST1 included the two pore channel *Tpcn1*; the Rho activating protein, *Abra,* which has been implicated in cardiac hypertrophy [55]; the kinase *Sik1;* and the uncharacterized genes *Gatsl2* and *Wdr75*.

To confirm our ChIP-seq results, we performed ChIP-qPCR validation on 17 novel TWIST1 binding sites using primers within the ChIP-seq peak regions. We used the known TWIST1 target *DNM3os* as a positive control (Figure 4B) [56] and the promoter region of *Foxa2* as a negative control. We found ChIP-qPCR enrichment for all novel TWIST1 bound regions analyzed as compared to the negative control. Significantly, our novel TWIST1 targets identified in the embryonic heart were also bound by TWIST1 in the developing limb suggesting that they are bound by TWIST1 in several cellular contexts. Taken together these results suggest that TWIST1 plays a critical role in endocardial cushion development, by directly regulating spatially and temporally restricted gene expression.

## Discussion

We have used a combination of Tag-seq and ChIP-seq to examine the *in vivo* functions of TWIST1 in the E10.5 developing mouse heart valves. By comparing genome-wide analysis of gene expression changes in the *Twist1* null AVC with spatial expression data in the E10.5 heart, we identified dramatic changes that occur in the AVC in the absence of TWIST1. Furthermore, using ChIP *in vivo* in the E10.5 heart, we identified 17 novel direct target genes of TWIST1 with expression changes in the *Twist1* mutant embryos.

### Identification of AVC- and OFT-enriched Genes

In this study, we describe the generation and analysis of five Tag-seq libraries from the atria, ventricles, OFT, and wild-type and *Twist1* null AVCs of the developing E10.5 mouse heart. These Tag-seq libraries were sequenced to a depth of over 7 million tags per library. Using these Tag-seq libraries we identified 725 genes enriched in both the AVC and OFT, which included 123 transcriptional regulators. The majority of AVC-enriched genes overlapped with the OFT-enriched genes, highlighting the shared mechanisms underlying the development of the valves in these two regions. However, there were some notable exceptions of genes enriched in the OFT but not the AVC, and vice versa, indicative of gene expression differences underlying unique AVC and OFT developmental processes. Although both the AVC and OFT form valves to control blood flow, the OFT is also critical in the formation of the main arteries leaving the left ventricle. Accordingly, Gene Ontology categorization identified vasculature development as enriched in the genes specific to the OFT and not the AVC. Similarly, enrichment of the category of neural crest cell differentiation highlights the role that neural crest cells play in the development of this tissue. Interestingly, the AVC was significantly enriched for genes involved in calcium ion homeostasis, whereas the OFT was not. The gene expression differences between the AVC and OFT could also reflect differences in timing of cushion formation, the cells populating these regions, or the cardiogenic lineage giving rise to them. For example, the most differentially expressed gene in the OFT was *Isl1*, a marker of the secondary heart field that contributes cells to the OFT and right ventricle [57], while the most highly AVC-specific gene was *Galanin*, a neuropeptide known to be expressed in the AVC before becoming restricted to AV-node and AV-rings [58].

The Wnt, TGFβ and Notch pathways have been shown to be critically involved in controlling endocardial cushion EMT and their activity must be tightly regulated [1], [10]. Modulators of these pathways were represented in our AVC- and OFT-enriched gene list (Table 2). *Csnk1e*, a casein kinase responsible for DISHEVELLED phosphorylation, and *Rspo3*, an inhibitor of Wnt receptor internalization, control β-CATENIN dependent transcriptional activation. Among the genes controlling TGFβ signalling we identified *Bmper*, a secreted factor that directly interacts with BMP ligands, and *Htra1*, a secreted serine protease that inhibits TGFβ family members by its proteolytic activity. Finally, enriched regulators of the Notch pathway included the bHLH transcription factor *Hes6*, and the Groucho related transcriptional co-repressors *Aes* and *Tle2*. The most highly expressed signaling pathway modulator in the AVC and OFT was *Igfbp5*, which has been shown to promote cartilage anabolism and osteoblast proliferation [59]. This is significant in light of research showing shared gene expression in developing heart valves, cartilage, bone and tendons [60].

Following EMT, newly formed mesenchymal cells undergo proliferation resulting in the expansion of the endocardial cushions. In our list of AVC- and OFT-enriched genes we identified several mediators of cell proliferation such as *Ccnd2* and *Cdca7*. Mesenchyme cells then undergo further differentiation characterized by expression of complex ECM molecules and matrix metalloproteinases. Previously characterized endocardial cushion ECM and structural proteins such as *Tenascin C*, *Perlecan (Hspg2)*, and several collagens (e.g. *Col3a1*, *Col9a3*, and *Col5a1*) were highly enriched in the AVC and OFT at E10.5. Significantly, our list of AVC- and OFT-enriched genes also contained novel endocardial cushion ECM proteins and modifiers such as the matrix metallopeptidase 14 (*Mmp14*).

### TWIST1 Regulates Gene Expression Patterns in the AVC

*Twist1* was the most highly expressed, DNA-binding, transcription factor in the E10.5 AVC where its expression is restricted to the mesenchyme cell population [20], [21]. Research in chick AVC development suggests a role for TWIST1 in the promotion of proliferation and migration of endocardial cushion cells, together with inhibition of their differentiation [23], while over-expression studies in mice implicated TWIST1 in ECM gene expression in the AVC [24]. However, no obvious morphological differences were observed in the *Twist1* null mouse AVC [26]. This apparent lack of AVC phenotype in the *Twist1* null might reflect a defect in maturation not seen before embryonic lethality at E11. Thus, we analyzed gene expression changes in the *Twist1* null phenotype by creating a Tag-seq library from the AVC of E10.5 *Twist1* mutant mice.

Comparison of *Twist1* null and wild-type AVC gene expression revealed remarkable changes consistent with a role for TWIST1 in determining proper AVC and OFT gene expression. Genes down-regulated in the *Twist1* null AVC were enriched in the wild-type AVC and OFT, while up-regulated genes were more likely to be enriched in atria and ventricles. This suggested that TWIST1 was directly or indirectly regulating many valve-specific genes, and in the absence of TWIST1, there was a shift in the cellular composition from an AVC phenotype to a more atria and/or ventricle-like phenotype. Genes down-regulated in the *Twist1* null AVC were enriched for cell migration and ECM molecules, while up-regulated genes were enriched in metabolic pathways and energy production. Cell migration is a critical step during EMT as the endocardial cells invade the cardiac jelly and TWIST1 has been associated with both the expression of promigratory genes and the proliferation of newly transformed cells [61]. TWIST1 has also been linked to the maintenance of energy and cell metabolism previously as it suppresses brown fat metabolism in adipose tissue [62]. In this context, over-expression of *Twist1* caused a reduction in mitochondria, while heterozygous *Twist1* mice were obesity resistant on a high-fat diet due to an increase in expression of oxidation genes. We observed that genes encoding several subunits for each of NADH dehydrogenase, ubiquinal-cytochrome c reductase, cytochrome c oxidase, and mitochondrial ATP synthase complexes were significantly up-regulated in the *Twist1* null AVC. These oxidative genes are critical in the electron transport chain and set up the cells to make ATP. Interestingly, many of the essential enzymes in the 10 reaction pathway of glycolysis, an alternate metabolic pathway used to form ATP, were also up-regulated.

Surprisingly, from the genes previously identified as TWIST1 targets in the chick AVC [23], [63] only *Periostin*, and to a lesser extend *Cdh11* and *Mmp2*, showed expression changes in the *Twist1* null AVC. Other members of the bHLH transcription factor family, such as TWIST2 [64], could compensate for lack of TWIST1 activity in the mouse. However, *Twist2* expression was substantially lower than *Twist1* expression and was not significantly altered in the null AVC.

Critical factors involved in valve development were also affected in the *Twist1* null mice. *Sox9, Sox4* and *Bmp2* were down-regulated significantly. In wild-type mice, *Sox9* is expressed, activated and required in the cushions following migration of the endocardial cells into the cardiac jelly in the cushions during EMT [65]. *Sox4* is expressed in the endocardium equally in both valve forming tissues, while *Bmp2* is normally expressed in the myocardium overlying the AVC at this stage of development [66], [67]. SOX9 is required for expansion of the precursor cell population early in valve development and later for proper expression of ECM proteins [13] and SOX4 is required for proper formation of the semilunar valves in the OFT [68]. Prior to cushion formation, BMP2 has two roles: it promotes cardiac jelly formation and it induces the overlying endocardium to undergo EMT [69]. As shown in chick, following EMT, BMP2 induces cell migration, but not proliferation, of mesenchymal cells and induces expression of *Periostin, Twist1* and *Id1* [70].

Using ChIP-seq we analyzed the ability of TWIST1 to bind to the regulatory sequences of our list of differentially expressed genes and identified a number of novel direct targets of TWIST1. Interestingly, we found that most genes differentially expressed between wild-type and *Twist1* null AVC did not have direct TWIST1 DNA-binding sites by ChIP-seq. However, our ChIP-seq was not saturating and many TWIST1 DNA-binding sites remain to be identified. We focused on high-confidence peaks, thus, further analyses may reveal additional direct targets. Of the 647 genes with TWIST1 bound as indicated by our limited ChIP-seq analysis, we found 57 were significantly mis-regulated in the *Twist1* null AVC and of these 30 were differentially expressed in the AVC. Genes whose regulatory sequences were bound by TWIST1 could be either up-regulated in the *Twist1* null AVC, such as *Tpcn1* and *Wdr75*, or down-regulated, such as *Tpm4* and *Chd9*. This indicated that TWIST1 can act both as an activator or inhibitor of transcription in the context of AVC development. Importantly, we validated 17 genes as novel direct targets of TWIST1.

Although there were few transcription factors identified here with evidence of TWIST1 binding and mis-regulation in the *Twist1* null AVC, changes in gene expression suggested that the lack of TWIST1 markedly changed the cells during development. It is evident by Tag-seq data that TWIST1 regulates expression of several critically important transcription factors in the AVC and is a central player in regulating AVC transcriptional networks.

## Supporting Information

Figure S1 **Dissection of E10.5 mouse heart.** E10.5 embryos were removed from pregnant females and their hearts were dissected using 18½G needles. Atria, ventricles, atrio-ventricular canals, and out-flow tracts were separated and collected in TRIzol reagent. The out-flow tract protrudes from the front of the heart and is outlined by the yellow dotted line. The red dotted line indicates the region collected as atrio-ventricular canal.
(DOC)

Figure S2 **RT-qPCR validation of AVC- and OFT-specific gene expression.** Tag-seq expression values for *Galanin*, *Adamts19*, *Isl1*, *Rgs5*, and *Gabra4* were validated by RT-qPCR across the atria, ventricles, atrio-ventricular canal (AVC), and out-flow tract (OFT). Graphs show relative quantification compared to ß-actin. Results are represented as average values from three independent samples ± standard deviations.
(DOC)

Table S1 **PCR Primers.** All primers used for PCR are listed. These include those used for *in situ* hybridization probe construction as shown in Figure 3, the ChIP-qPCR as shown in Figure 4 and the RT-qPCR as shown in Figure S2.
(XLS)

Table S2 **Gene expression of all genes in E10.5 heart Tag-seq libraries.** All genes expressed in E10.5 wild-type (atria, ventricles, atrio-ventricular canal (AVC), and out-flow tract (OFT)) and *Twist1* null AVC Tag-seq libraries. All expression values are represented as tags per million and only genes with more than 5 raw tags were considered expressed.
(XLS)

Table S3 **Expression of genes previously characterized in AVC development.** 16 genes previously identified as involved in AVC development were analyzed for AVC expression. The majority of these genes showed AVC enrichment. Expression values are listed for each library including the *Twist1* null AVC and are represented as tags per million. Only genes with more than 5 raw tags were considered expressed. Fold changes and p-values are provided as calculated by edgeR as the normalized fold-change of AVC expression over the atria and ventricle libraries.
(XLS)

Table S4 **Genes significantly enriched in the AVC and OFT.** Genes enriched in both the AVC and OFT (725 genes in Table S4A), just the AVC (214 genes in Table S4B) or just the OFT (324 genes in Table S4C) are listed. A heat-map of the data is shown in Figure 1. Expression values are listed for each library including the *Twist1* null AVC and are represented as tags per million. Only genes with more than 5 raw tags were considered expressed. Fold changes and p-values are provided as calculated by edgeR as the normalized fold-change of AVC expression or OFT expression over the atria and ventricle libraries. Genes were considered enriched if the p-value was less than 0.05 but no fold change cut-off was used.
(XLS)

Table S5 **Enriched Gene Ontology biological process categories in genes enriched in the AVC and OFT.** Gene Ontology (GO) analysis identified categories enriched in the 725 genes enriched in both the AVC and OFT (Table S5A), the 214 genes enriched in the AVC but not the OFT (Table S5B), and the 324 genes enriched in the OFT but not the AVC (Table S5C). GO analysis was done in DAVID: enrichment was calculated against the whole RefSeq database as background and p-values represent one-tail Fisher Exact Test statistics. Category titles, the number of genes represented in each category (count), the p-values and the Benjamini corrected p-values are provided for each category in addition to the list of genes in that category and the expression data. Only relevant and non-redundant categories were included in the table. Gene expression values are listed for each library including the *Twist1* null AVC and are represented as tags per million. Only genes with more than 5 raw tags were considered expressed. Fold changes and p-values are provided as calculated by edgeR as the normalized fold-change of AVC expression or OFT expression over the atria and ventricle libraries.
(XLS)

Table S6 **Altered gene expression in the** ***Twist1*** **null AVC.** Table S6A identifies the 283 genes down-regulated in the *Twist1* null AVC library while Table S6B identifies the 621 genes up-regulated in the *Twist1* null AVC library. A heat-map of the data is shown in Figure 2. Expression values are listed as tags per million for each library and only genes with more than 5 raw tags were considered expressed. Fold changes and p-values are provided as calculated by edgeR as the normalized fold-change of *Twist1* null AVC over wild-type (WT) AVC expression. Also included are AVC and OFT fold changes and p-values. Genes were considered up- or down-regulated if the p-value was less than 0.05 but no fold change cut-off was used.
(XLS)

Table S7 **Enriched Gene Ontology biological process categories in genes with altered expression in the** ***Twist1*** **null AVC.** For the 283 genes down-regulated (Table S7A) and the 621 genes up-regulated (Table S7B) in the *Twist1* null AVC, we identified enriched GO categories by using DAVID. Enrichment was calculated against the whole RefSeq database as background and p-values represent one-tail Fisher Exact Test statistics. Category titles, the number of genes represented in each category (count), the p-values and the Benjamini corrected p-values are provided for each category in addition to the list of genes in that category and the expression data. Only relevant and non-redundant categories were included in the table. Expression values are listed as tags per million for each library and only genes with more than 5 raw tags were considered expressed. Fold changes and p-values are provided as calculated by edgeR as the normalized fold-change of the *Twist1* null AVC over wild-type (WT) AVC. Also included are AVC and OFT fold changes and p-values.
(XLS)

Table S8 **Genes significantly differentially expressed in the** ***Twist1*** **null AVC with high confidence TWIST1 ChIP-seq peaks.** Genes with a TWIST1 ChIP-seq peak of height 30 or more were compared to genes differentially expressed in the *Twist1* null AVC. 26 genes were down-regulated (Table S8A) and 31 were up-regulated (Table S8B) and had at least one TWIST1 peak. Peaks were associated with genes using GREAT and the genomic location of the peaks and the peak heights are listed. Expression values are listed as tags per million for each library and only genes with more than 5 raw tags were considered expressed. Fold changes and p-values are provided as calculated by edgeR as the normalized fold-change of *Twist1* null AVC over wild-type (WT) AVC expression. Also included are AVC and OFT fold changes and p-values. Genes were considered up- or down-regulated if the p-value was less than 0.05 but no fold change cut-off was used.
(XLS)
