# Supplementary figures and images for: Twist1 Transcriptional Targets in the Developing Atrio-Ventricular Canal of the Mouse
Source: PLoS One. 2012 Jul 16;7(7):e40815. doi: 10.1371/journal.pone.0040815 (PMC3397961; doi:10.1371/journal.pone.0040815)

**Figure S1. Dissection of E10.5 mouse heart**


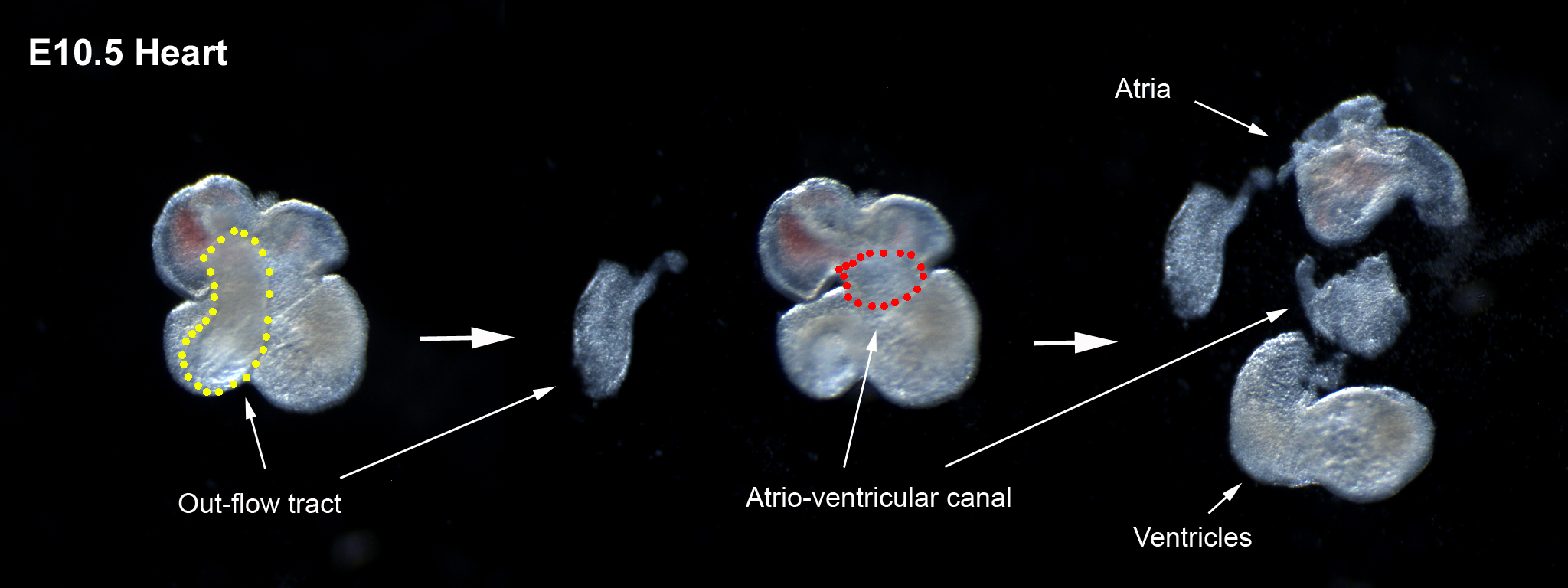

Supplement: Figure S1 — Dissection of E10.5 mouse heart. E10.5 embryos were removed from pregnant females and their hearts were dissected using 18½G needles. Atria, ventricles, atrio-ventricular canals, and out-flow tracts were separated and collected in TRIzol reagent. The out-flow tract protrudes from the front of the heart and is outlined by the yellow dotted line. The red dotted line indicates the region collected as atrio-ventricular canal. (DOC) [file pone.0040815.s001.doc]

**Figure S2. RT-qPCR validation of AVC- and OFT-specific gene expression**

**
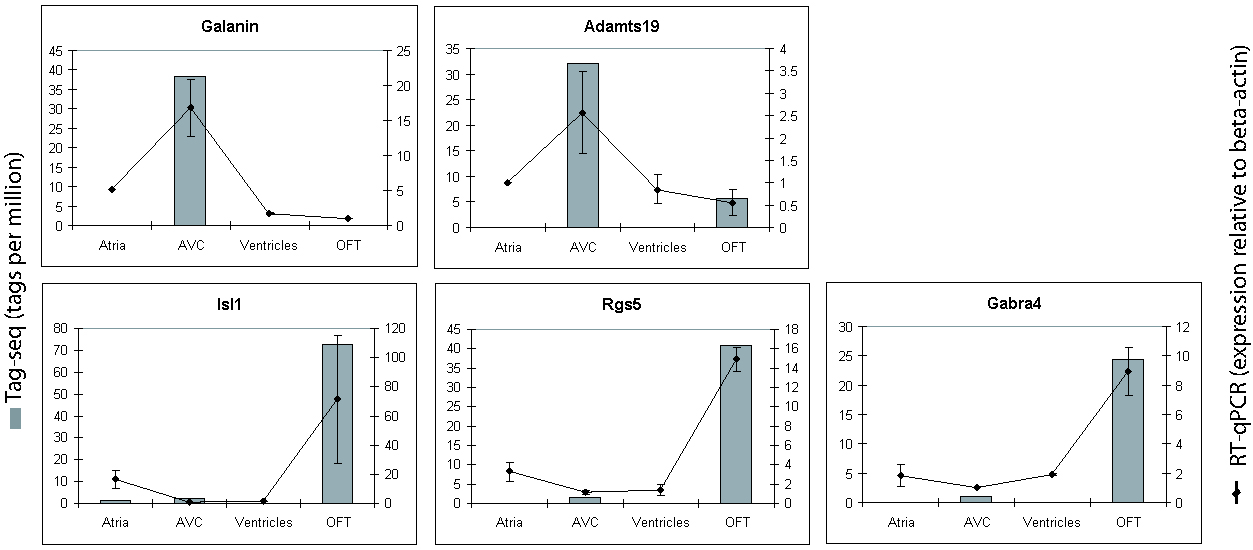
**

Supplement: Figure S2 — RT-qPCR validation of AVC- and OFT-specific gene expression. Tag-seq expression values for Galanin, Adamts19, Isl1, Rgs5, and Gabra4 were validated by RT-qPCR across the atria, ventricles, atrio-ventricular canal (AVC), and out-flow tract (OFT). Graphs show relative quantification compared to ß-actin. Results are represented as average values from three independent samples ± standard deviations. (DOC) [file pone.0040815.s002.doc]
